# Triple Pancake Bonding in Neutral π‑Conjugated Dimers: a Computational Study

**DOI:** 10.1021/jacs.6c07884

**Published:** 2026-07-02

**Authors:** Li-juan Cui, Miklos Kertesz, Zhong-hua Cui

**Affiliations:** † Institute of Atomic and Molecular Physics, 12510 Jilin University, Changchun 130023, China; ‡ Department of Chemistry and Institute of Soft Matter, 8368 Georgetown University, 37th and O Streets, NW, Washington, D.C. 20057, United States; § Key Laboratory of Physics and Technology for Advanced Batteries (Ministry of Education), Jilin University, Changchun 130023, China

## Abstract

High-order pancake bonding arising from the overlap of multiple π-type orbitals in π-conjugated molecules is exceedingly rare. Recently reported cofacially stacked hexaazatrinaphthylene trianions ([HAN]^3–^) stabilized by tetravalent actinides exhibit six-electron triple pancake bonds, but the presence of counterions hides the intrinsic nature of the bonding. Here, we designed a neutral HAN derivative via hydrogen coordination, 1,5,9-trihydro-1,4,5,8,9,12-hexaazatriphenylene (HATH_3_), which features a quartet ground state with three π-type singly occupied molecular orbitals. The HATH_3_ monomer dimerizes both in *trans*- and *cis*-cofacial arrangements, with ultrashort intermolecular separation of 2.968 and 2.971 Å with substantial interaction energy of −158.6 and −135.1 kJ/mol, respectively. The stability of these dimers occurs primarily through orbital interactions, three electron-sharing π-orbitals between two HATH_3_ fragments. Electrostatic interactions and dispersion make smaller but significant bonding contributions to the overall stability. These neutral dimers exhibit a genuine triple pancake bond, providing new insight into the nature of high-order π-stacking interactions. These strong intermolecular interactions can be important in aggregate formation and crystal formation.

## Graphical abstract

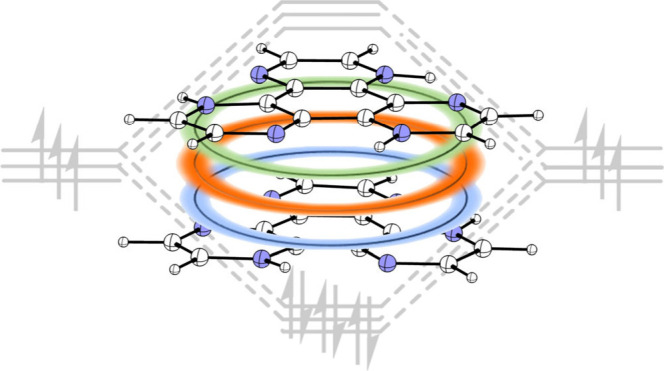

## Introduction

Planar stable π-conjugated organic radicals, together with their strong π–π stacking ability, are fundamental building blocks in a wide range of organic materials.^1–3^ A distinct mode of intermolecular π-stacking observed in radical dimers is pancake bonding, which is characterized by intermolecular contact distances along the stacking direction that are shorter than typical van der Waals separations.^4–6^ Pancake bonding arises from the direct overlap of highly delocalized π-type singly occupied molecular orbitals (SOMOs) typical for radicals, leading to bonding interactions with strong orientational preferences and partial covalent character.^6,7^ This type of intermolecular interaction provides an atom-over-atom π-stacking configuration, as opposed to the typical intermolecular packing between closed shell molecules.^8–10^ The prototypical phenalenyl dimer, PLY_2_, exemplifies this phenomenon, where SOMO–SOMO overlap results in an atom-over-atom stacking geometry and a pancake bond order of one, FPBO = 1 (Scheme 1a).^11–13^ Crystalline systems featuring single pancake bonds, such as phenalenyl derivatives,^14–17^ tetracyanoquinodimethane (TCNQ),^18–21^ tetracyanoethylene (TCNE),^22^ 1,2,4,6-thiatriazine (TTA),^23^ and related neutral and charged radicals, have been extensively investigated and show promise for applications in spin-mediated molecular functionalities, molecular conductors,^24–26^ and systems exhibiting magneto-, optical-, and electronic-property changes upon phase transitions.^27–30^ Notably, their unique intermolecular interactions, stacking motifs, and structure–property relationships have been extensively elucidated through a variety of theoretical approaches.

**Scheme 1:**
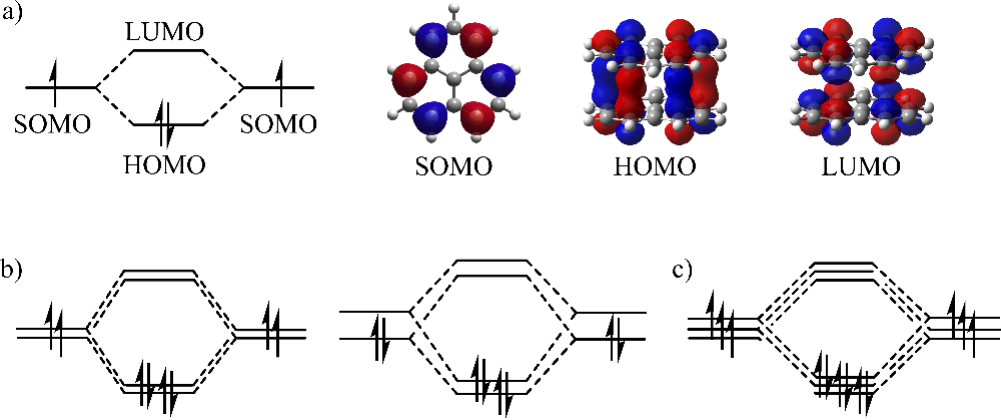
Molecular Orbital Diagrams for Different Orders of Pancake Bonding: a) Single Pancake Bond in the PLY_2_ π-Dimer Formed by Bonding (HOMO) and Antibonding (LUMO) Combinations of Two SOMOs, FPBO = 1; b) Double Pancake Bonding Based on a Diradical or a Closed Shell Monomer with a Small HOMO-LUMO Gap, (FPBO = 2); c) Triple Pancake Bonding Formed by the Interaction of Multiple SOMOs (FPBO = 3)

In principle, molecular orbital interaction diagrams indicate that pancake bonding can be extended to higher orders.^6^ In particular, a double pancake bonding mechanism is expected to emerge from interactions between π-type orbitals in systems possessing either a singlet ground state with a low-lying triplet excited state or a triplet ground state (Scheme 1b). However, such electronic configurations are highly sensitive and difficult to stabilize. In 2014, Kertesz et al. introduced the concept of double pancake bonding to rationalize the unusually short inter-ring separation observed in crystalline 1,3,2,4,6-dithiatriazine (DTTA).^31^ In the same study, double pancake-bonded dimers of Se_2_N_3_CH, S_3_N_3_^+^ and C_5_H_5_^+^ were theoretically proposed, pushing the limits of strong covalent-like π–π interactions in terms of both exceptionally short intermolecular distances and substantial bonding energies. Subsequently, boron- and nitrogen-substituted phenalenyl dimers were suggested as additional candidates exhibiting double pancake bonding,^32^ while theoretical investigations of triangular graphene dimers predicted that pancake bond orders as high as five could be achievable.^33^ Despite these advances, high-order pancake-bonded systems remain exceptionally rare, and experimentally validated examples beyond the double-pancake bond regime are still scarce.

Very recently, Layfield et al. reported the synthesis of a three-electron-reduced hexaazatrinaphthylene (HAN) and the subsequent stacking of the resulting [HAN]^3–^ triradicals (Scheme 2).^34^ In this system, tetravalent thorium and uranium ions stabilize the strong electrostatic repulsion between the negatively charged monomers. Notably, this work represents the first experimental realization of triple pancake bonding, characterized by three π-type SOMO–SOMO overlaps, as illustrated in Scheme 1c. The stabilizing role of metals in the complexes of Layfield et al. enabling the SOMO–SOMO bonding interactions was clearly established in ref 34. In these complexes the HAN units can be characterized by a formal charge of −3. We designed a neutral HAN derivative, termed HATH_3_ (Scheme 2), generated via hydrogen coordination at nonadjacent nitrogen sites of 1,4,5,8,9,12-hexaazatriphenylene (HAT), the core framework of HAN, transforming it into an electron rich neutral analogue of HAT with a formal π-electron count of 21, the same as that of the three-electron-reduced HAN, [HAN]^3–^.^35,36^ Neutral HATH_3_ adopts *C*_3*h*_ symmetry and stabilizes a quartet ground state, in which three π-type singly occupied molecular orbitals are delocalized over the three pyrazine branches (Scheme 2d). Similar to [HAN]^3–^, HATH_3_ therefore exhibits a strong propensity to form triply pancake-bonded assemblies. Structural analysis reveals that a *trans*-cofacial arrangement of HATH_3_ constitutes a true minimum with favorable thermodynamic stability. This system thus represents the rare example of a neutral triply pancake-bonded assembly.

**Scheme 2:**
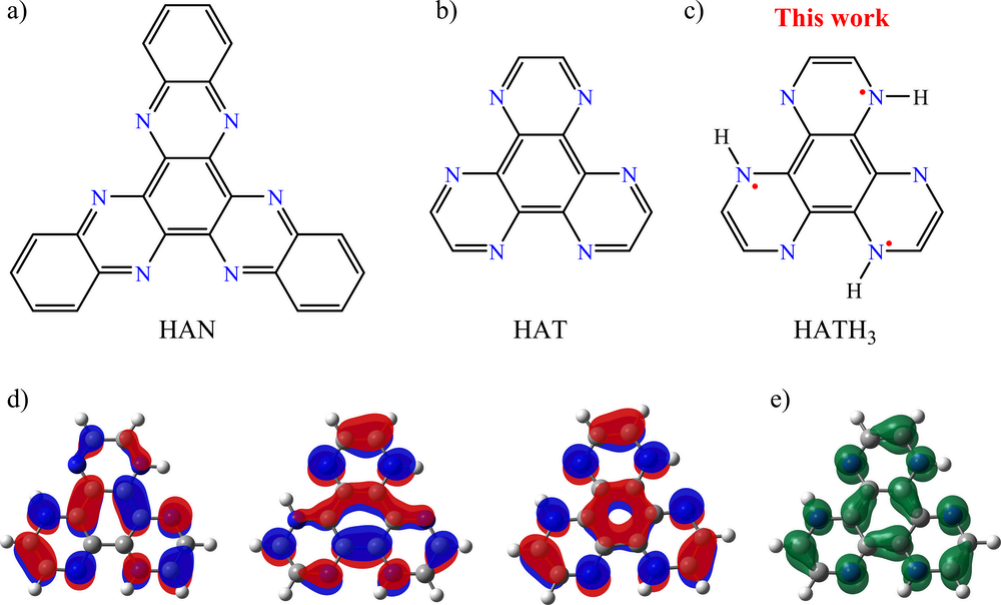
Chemical Structures of a) HAN (Hexaazatrinaphthylene), b) HAT (1,4,5,8,9,12-Hexaazatriphenylene), and c) HATH_3_ (1,5,9-Trihydro-1,4,5,8,9,12-hexaazatriphenylene); d) Diagram of the Three Nearly Degenerate SOMOs of HATH_3_, and e) Total Unpaired Spin Density of HATH_3_

Triphenylene (TPh)^37^ can be considered as the parent molecule to HAT and HATH_3_. This connection explains some of the similarities in their electronic structures, and unsurprisingly, there are related pancake-bonded oxidized salts of TPh in the literature.

Both TPh and HAT are closed-shell molecules. Their MO diagrams are provided in Figure S1. While TPh is an alternant hydrocarbon with a well-known electron–hole symmetry, HAT, its derivative is not.^35^ Both display doubly degenerate HOMOs and LUMOs, which can serve as hosting unpaired electrons that in turn can contribute to pancake bonding. In the case of TPh, it has been observed that in its salts due to oxidation, TPh loses electrons, which in turn produce pancake-bonded aggregates. The presence of unpaired electrons in HATH_3_ is accomplished by the additional π-electrons as the pyridine-type sp^2^ nitrogen atoms are converted into pyrrole-type sp^2^ nitrogen atoms. The additional three π-electrons occupy the LUMO and LUMO+1 orbitals, one of which is degenerate, and all three are nearly degenerate. Interestingly, the respective three LUMO and LUMO+1 orbitals are near but not nearly degenerate in TPh. A similar situation of near degeneracy is present in the orbital pattern of HATH_3_CN and also in HANH_3_. Consequently, all three (HATH_3_, HATH_3_CN, and HANH_3_) have a high-spin ground state with S = 3/2, as confirmed at the SC-NEVPT2 level (Table S1), which turns out to be the basis of the presented triple pancake bonding.

The experimental literature shows some pancake bonding in dimers and trimers of partially positively charged TPhs. These experimental findings are summarized below. The first mention of close aggregation of triphenylene radical cation dimers was identified in (C_18_H_12_)_2_^•+^(PF_6_^–^) and (C_18_H_12_)_2_^•+^(AsF_6_^–^), constituting conducting solids state salts; unfortunately, without a detailed X-ray diffraction-based structural refinement.^38^ Pancake-bonded dimers of TPh are found in a complex clathrate (CSD refcode: FARFIO). This is a highly disordered structure where the charge assignment is ambiguous.^39^ The pancake-bonded dimers of TPhs are more precisely identifiable in their charge transfer salt with tetrafluoro-tetracyanoquinodimethane (CSD refcode: PAYBAW).^40^ The composition is [2­(C_18_H_12_)]^+^[C_12_F_4_N_4_]^−^. The crystal contains pancake-bonded dimers of TPh, each with a formal charge of +1/2. The latest single crystal structure of pancake-bonded trimers of TPh (CSD refcode: DOYSUJ)^41^ corresponds to a formal charge of +1/3 positive charge each with the overall chemical repeat unit of [(C_18_H_12_)_3_]^•+^(Ga_3_Cl_10_)^−^. This experimentally observable richness of the ability of the parent molecule, TPh, to produce pancake bonding supports the idea that pancake-bonded aggregates of the presented neutral high spin molecules will be observable.

We present a comprehensive intermolecular interaction analysis identifying which orbital interactions are responsible for this unprecedented form of strong through-space π-bonding for three derivatives of TPh, providing new insights into the design and discovery of multiply pancake-bonded molecular systems. These strong intermolecular interactions can be important in crystal and aggregate formation.^42–44^

## Results and Discussion

### Validating DFT Methods

Pancake-bonded systems require a rigorous treatment of both static electron correlation, arising from quasi-degenerate frontier orbitals, and dynamic electron correlation associated with dispersion interactions.^6,31^ High-level multireference methods, such as multireference averaged quadratic coupled cluster (MR-AQCC),^45^ provide a balanced description of static and dynamic correlation and yield reliable geometries and interaction energies for pancake-bonded systems. However, for the systems studied here, the large molecular size and extensive active spaces render MR-AQCC calculations computationally prohibitive. As a practical alternative, spin-unrestricted density functional theory with the broken-symmetry approach (UDFT)^46^ offers a reasonable compromise between accuracy and computational cost for describing both geometrical structures and intermolecular interactions, as demonstrated in previous studies. Accordingly, we selected (PLY)_2_,^13^ (TTA)_2_,^31^ and (DTTA)_2_ ^31^ as benchmark models shown in Figure S2, representing systems with single and double pancake bonds, respectively. Four functionals (UM05–2X,^47^ UM06L,^48^ UPBE0-D3,^49^ and UB3LYP-D3 ^50^) were evaluated in comparison with MR-AQCC reference data. Deviations between the UDFT and MR-AQCC results are quantified, with key energetic and structural parameters summarized in Figure S3 and S4 and Table S3. Consistent with the findings of Mou et al.,^51^ our results confirm that UM05–2X performs exceptionally well in describing the total interaction energies (E_int_) for the pure hydrocarbon PLY_2_ system. However, it performs less accurately in characterizing SOMO–SOMO interaction energies (E_SOMO–SOMO_) compared to the UM06L, UPBE0-D3, and UB3LYP-D3 methods. For stronger orbital coupling systems such as TTA_2_ and DTTA_2_, most methods struggle to reproduce the SOMO–SOMO interaction energy (E_SOMO–SOMO_) calculated by the MR-AQCC method, except for UM06L and UB3LYP-D3. Regarding the dispersion effect, our supplemental tests confirmed that the D3 correction provided no improvement for the UM06L theory. Therefore, the uncorrected UM06L method was adopted to avoid artificial overbinding. Consequently, to ensure a comprehensive and rigorous description of the triple pancake bonding in the (HATH_3_)_2_ system, the UM06L method was employed for all energy and bonding analyses in this work.

### Geometric Features and Stability Analysis of the (HATH_3_)_2_ Dimers

As shown in Figure 1, the designed two π-dimers, the *trans*- and *cis*-cofacial arrangements of (HATH_3_)_2_, were predicted as local minima with the two lowest vibrational frequencies of 64.4 and 48.3 cm^–1^, respectively, at the UM06L/6–311++G­(2d,2p)^52^ level. The *trans* π-dimer is 23.5 kJ/mol more stable than the *cis* π-dimer. The *trans* π-dimer adopts *D*_3_ symmetry with a singlet ^1^A_1_ ground state, exhibiting a small torsional angle of 8.0° between two monomers around the *C*_3_ axis. This geometry allows the central carbon atoms to achieve a nearly perfect atom-over-atom configuration, while the peripheral hydrogen atoms can better avoid steric crowding. A similar slight intermolecular torsion is seen in the dimers of HATH_3_CN and HANH_3_ as discussed at the end of this work. In contrast, the *cis* π-dimer features two monomeric units stacked face to face, retaining *C*_3*h*_ symmetry with ^1^A’ symmetry in the ground state. The vertical separations between the central C_6_ rings in the *trans* and *cis* π-dimer are 2.968 and 2.971 Å, respectively, markedly shorter than twice the van der Waals radius of carbon (3.40 Å)^53^ and the interlayer distance of 3.35 Å in graphite.^54^ The intermolecular distances of the *trans* and *cis* π-dimers are detailed in Table S4. To evaluate the stability of the stacked dimers, we performed rigid potential energy surface (PES) scans by displacing one HATH_3_ monomer along the *x* and *y* axes while maintaining the equilibrium interplanar distance at UM06L/6–311++G­(2d,2p) level (Figure S5). For both *trans* and *cis* π-isomers, the energy rises sharply within a displacement of 1.0 Å, reaching barriers of approximately 126 kJ/mol. At larger displacements, the relative energy curve exhibits oscillations, corresponding to the periodic alternation between interatomic repulsion and orbital overlap as the monomer frameworks slide past one another.

**Figure 1:**
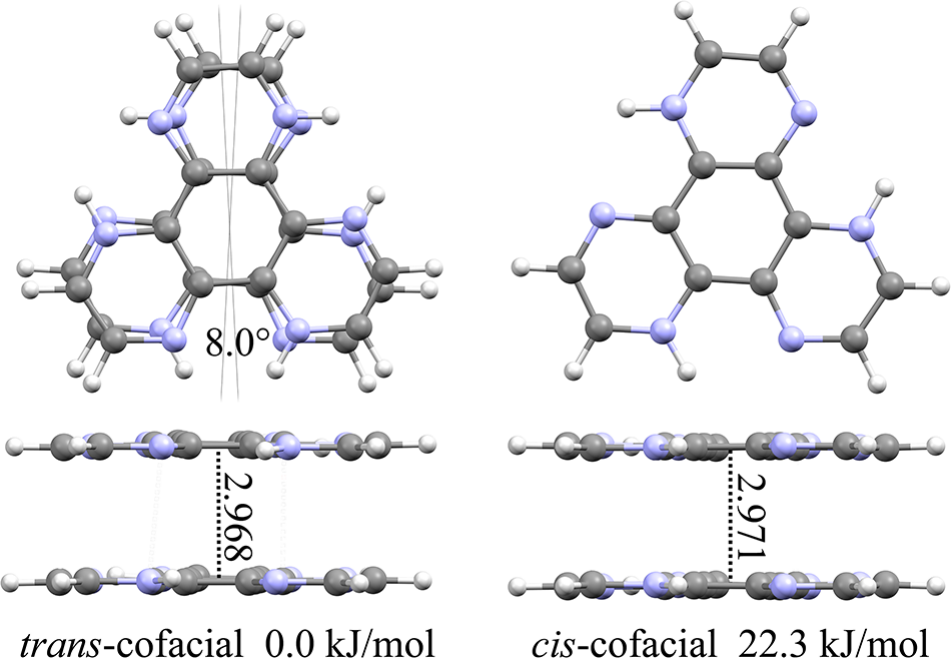
Geometries and relative energies of the (HATH_3_)_2_ π-dimer at the UM06L/6–311++G­(2d,2p) level. The vertical separations between the central *C*_6_ rings are expressed in angstroms (Å).

The interaction energy, E_int_, is defined as the energy difference between the dimeric complex and its constituent isolated monomers. For the π-dimers, E_int_ is approximately expressed as a sum of two components, SOMO–SOMO interaction energy (E_SOMO–SOMO_) and the remaining van der Waals term (E_vdw_), as expressed by eqs 2 −5.^13,55,56^ Within this framework, E_SOMO–SOMO_ represents the bonding component between the partially occupied orbitals, while E_vdw_ includes dispersion, Pauli repulsion, and electrostatic terms obtained from the computation of the high spin state of the dimer as discussed in the works by Mota et al. and Cui et al. as well as in the methods section. This approximate decomposition of the interaction energy assumes that the ground state of the dimer is the low spin state, which is the case here. The respective interaction energies and their components of the *trans* and *cis* π-dimers in different spin states using the singlet equilibrium geometry are collected in Table 1. In the singlet minimum of the *trans*, E_SOMO–SOMO_ reaches −213.8 kJ/mol at the UM06L/6–311++G­(2d,2p), more than compensating for the strong vdW repulsion and yielding a large net stabilization of −158.6 kJ/mol. The E_int_ for the septet state is repulsive with +55.2 kJ/mol, which is used to approximate E_vdW_ according to eq. 4. The *cis* π-dimer exhibits a slightly stronger SOMO–SOMO interaction than the *trans* π-dimer, yet a reduced overall stabilization due to significantly larger van der Waals repulsion (+80.3 kJ/mol) imposed by its perfectly cofacial stacking geometry. The calculated spin expectation values (Table 1) match theoretical ideal values, confirming that our results are nearly free from spin contamination and electronically reliable. The coexistence of such strikingly short interfacial distances and high stability provides strong evidence for the existence of multiple pancake bonding within these (HATH_3_)_2_ dimers.

**Table 1:** Interaction Energy Components (kJ/mol) and Spin Expectation Values ⟨S^2^⟩ for (HATH_3_)_2_ π-Dimers at the UM06L/6-311++G­(2d,2p) Level^a^

	E_int_	E_SOMO–SOMO_	E_vdW_	⟨S^2^⟩
*trans* π-dimer				
Singlet	–158.6	–213.8	55.2	0.0000
Septet	55.2	0.0	55.2	12.0460
*cis* π-dimer				
Singlet	–135.1	–215.9	80.3	0.0000
Septet	80.3	0.0	80.3	12.0428

a Calculations were performed for different spin states at the singlet ground state geometry (see eq 2–5).

We further employed various UDFT methods to compute the interaction energies and their components of the *trans* π-dimer (Table S5). Ultimately, these consistent results across multiple levels of theory confirm that the stabilization of the *trans* π-dimer primarily originates from the SOMO–SOMO interaction. Among the hybrid functionals, a distinct trend emerges as the HF exchange percentage decreases: the E_SOMO–SOMO_ interaction increases in magnitude while the E_vdW_ repulsion decreases. However, as noted by Mou et al., the high HF exchange in UM05–2X is meticulously balanced with other density functional terms, allowing it to accurately reproduce total interaction energies for systems like the PLY_2_. For the *trans* π-dimer of (HATH_3_)_2_, UM05–2X yields an E_int_ of −46.9 kJ/mol with an intermolecular distance of 2.995 Å. Compared to UM06L, UM05–2X predicts a larger separation, which inherently leads to a lower E_int_ and a reduced E_SOMO–SOMO_ contribution of −110.5 kJ/mol. For the *cis* (HATH_3_)_2_ π-dimer, UM05–2X predicts an even larger intermolecular distance of 3.192 Å. At this separation, although the *cis*-isomer maintains a perfectly cofacial overlap, the decreased orbital coupling at larger distances results in a notably weaker overall stabilization compared to the *trans*-isomer.

We then addressed possible dimer structures here and obtained two π-dimers and six single-bonded σ-dimers of HATH_3_ (Figure 2). The selection of the six (HATH_3_)_2_ σ-dimer configurations was strategically based on the distribution of SOMO density at the peripheral carbon and nitrogen sites, covering various C–C, C–N, N–N bonding patterns. It is important to clarify that our analysis focuses exclusively on these single-bonded rather than multiple bonded σ-species. Although certain multiple bonded σ-isomers might theoretically exhibit lower absolute energies, their formation requires drastic structural distortion of the rigid HATH_3_ framework and a complete sacrifice of the monomeric aromatic stabilization. Such extensive geometric reorganization imposes a prohibitive barriers under experimental conditions. All C–C, C–N σ-dimers are significantly less stable here, with the lowest-energy σ-dimer lying more than 70 kJ/mol above the π-dimer minimum. Furthermore, our calculations indicate that N–N σ-bonding patterns show a strong propensity to relax into π-dimer configurations during structural optimization. This behavior is attributed to the severe lone-pair repulsions between the nitrogen atoms, which destabilize a localized N–N σ-linkage. It is worth noting that the intermonomer σ-bonds in these σ-dimers are longer than typical single C–C (C–N) σ-bonds with bond distances at about 1.6 Å. The overall crowding of the immediate environments of the HATH_3_ to HATH_3_ σ-bonds explains their slight elongation relative to the standard CC and CN single σ-bonds and the fact that the σ-bonded species is less stable than the pancake-bonded dimers.

**Figure 2:**
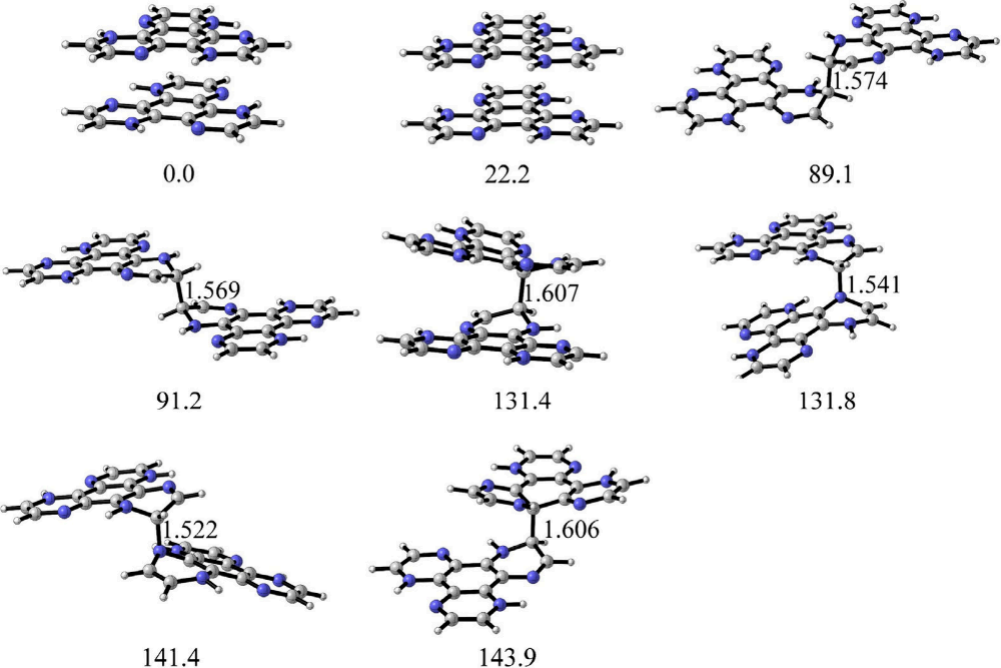
Computed relative energies (kJ/mol) of various π- and σ-dimers of HATH_3_ at UM06L/6–311++G­(2d,2p) level. Intermonomer σ-bond lengths (*r*, in Å) are shown.

### Electronic Structure of the (HATH_3_)_2_ π-Dimers

To accurately describe the electronic structure of (HATH_3_)_2_ π-dimers, we also employed the multiconfigurational CASSCF­(6,6) method at the UM06L optimized geometry, which is essential to account for the static correlation and to verify the multireference character of the pancake bonding. The orbitals have been obtained using the bonding and antibonding orbitals of the three π-type SOMOs in HATH_3_ as the active orbital space. Frontier molecular orbitals (FMOs) and corresponding natural orbital occupation numbers (NOONs)^57^ of the *trans* and *cis* π-dimers are shown in Figures 3 and S6. Three doubly occupied frontier orbitals of the *trans* π-dimer are comprised of bonding overlap of three fragmental SOMOs, which are highly delocalized. Furthermore, the complementary distributions of the SOMOs ensure that the intermolecular orbital overlaps for the three bonding orbitals are located on distinct atomic sites. Consequently, all six SOMO associated electrons in the dimer participate in this multicenter bonding (commonly denoted as 6e/mc bonding). Formal electron counting of three (slightly) bonding combinations of these SOMOs across the van der Waals gap leads to the notion of triple π–π bonding that covers all skeletal carbon and nitrogen atomic sites on both HATH_3_ rings, leading to the notion of a genuine triple pancake-bonded π-dimer.

**Figure 3:**
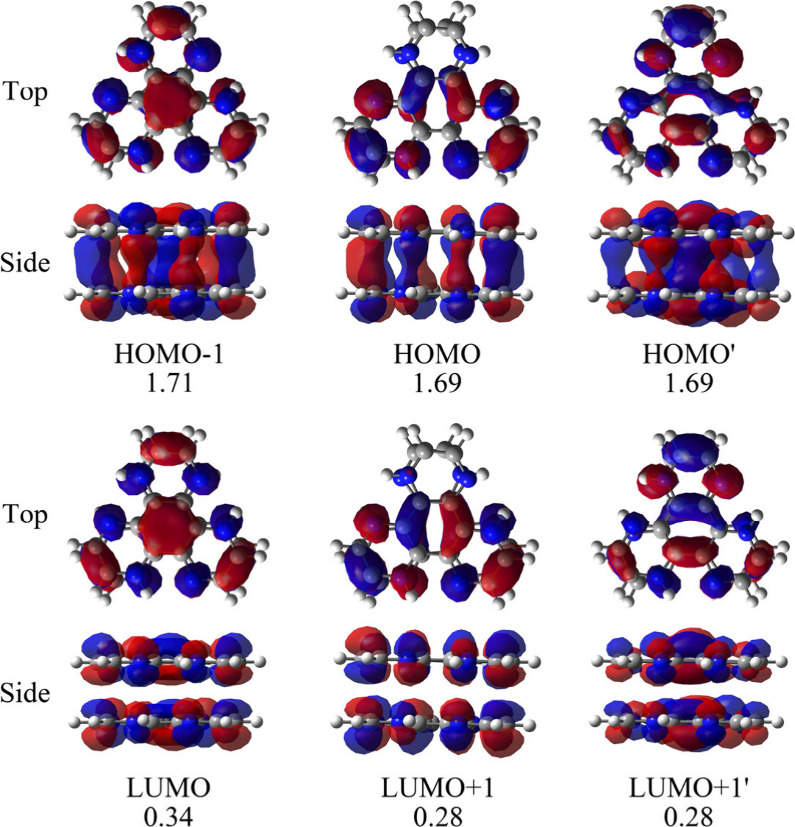
Frontier molecular orbitals of *trans* (HATH_3_)_2_ π-dimer at CASSCF­(6,6)/6–311++G­(2d,2p). Each orbital describing the pancake triple bonds is shown top-down and side-on. NOON values are also shown.

Physically reasonable measures of bond strength for such radical systems are derived from the NOONs based on six frontier orbitals, rather than relying on formal integer bond orders. To quantitatively assess the electronic coupling between the monomers, we calculated the effective bond orders (*p*_NO_) for the π–π interactions:

1 $$p_{NO}=\left(\mathrm{NEBO}-\mathrm{NEABO}\right)/2$$

where NEBO is the number of electrons in bonding orbitals and NEABO is the number of electrons in the antibonding orbitals based on the natural orbital occupancies for the six frontier orbitals. The *p*_NO_ values obtained are 2.104 for *trans* and 2.099 for *cis*. This observation is as expected due to the triradicaloid quartet ground state of HATH_3_.

### EDA-NOCV Analysis of the Triple Pancake Bond

To obtain a quantitative picture of the bonding energetics, we further adopted an energy decomposition analysis (EDA) coupled with the natural orbital for chemical valence (NOCV) theory.^58,59^ Two neutral HATH_3_ molecules in their quartet state, which is their ground state, were considered as the interacting moieties.

Table 2 shows the numerical results of the EDA-NOCV calculations. As expected, the dominant stabilizing contribution to the pancake bond formation arises from the orbital interactions (ΔE_orb_), which account for approximately 60% of the total attraction. This primary role of ΔE_orb_ underscores the covalent-like nature of the intermolecular bonding (HATH_3_)_2_ dimer, distinguishing it from conventional dispersion-driven π-stacking. Electrostatic interactions (ΔE_elstat_) contribute ∼40%. Notably, the Pauli repulsion is significant at ΔE_Pauli_ = 210.5 kJ/mol for *trans*, which is a direct consequence of the exceptionally short stacking distance (2.97 Å). Although the *trans*-dimer exhibits higher Pauli repulsion than the *cis*-dimer, its superior electrostatic stabilization more than compensates for this penalty, securing its overall thermodynamic preference. Regarding the treatment of dispersion, the Minnesota functionals already contain some amount of dispersion by parametrization. The stabilizing effects of intermolecular dispersion are inherently encapsulated within the interaction energy. We avoided adding explicit Grimme-type dispersion corrections to prevent artificial overestimation of the interaction energy. We further resolved these physical components of ΔE_int_ using the UPBE0-D3 level of theory, which partitions dispersion explicitly (Table S6). The ΔE_elstat_ contribution remains consistent at approximately 40% of the total attraction, ΔE_orb_ is comparable to the electrostatics, accounting for another 40%, while the explicit dispersion term (ΔE_disp_) provides the remaining 20%.

**Table 2:** EDA Results of the (HATH_3_)_2_ π-Dimer Calculated at the UM06L/TZ2P-ZORA Level Using the UM06L/6-311++G­(2d,2p) Geometry, Considering the Interaction between Two Neutral HATH_3_ Molecules in Their Ground State, Which Is a Quartet (Energy Values Are Given in kJ/mol)

Energy	Interaction	*trans*	*cis*
Δ*E*_int_		–150.6	–128.9
Δ*E*_hybrid_ ^a^		96.2	95.0
Δ*E*_Pauli_		210.5	194.1
Δ*E*_elstat_ ^b^		–142.3 (39.4%)	–109.2 (33.8%)
Δ*E*_orb_ ^b^		–218.8 (60.6%)	–213.8 (66.2%)
			
Δ*E*_orb(1)_ ^c^	electron-sharing π bond (A_1_)	–65.7 (30.0%)	–63.6 (29.7%)
Δ*E*_orb(2)_ ^c^	electron-sharing π bond (E)	–61.1 (27.9%)	–61.1 (28.6%)
Δ*E*_orb(3)_ ^c^	electron-sharing π bond (E)	–61.1 (27.9%)	–61.1 (28.6%)
Δ*E*_orb(rest)_ ^c^		–31.0 (14.2%)	–28.0 (13.1%)

a Metahybrid correction toward orbital interaction.

b The percentage contribution with respect to total attraction is given in parentheses.

c The percentage contribution in parentheses is given with respect to total orbital interaction.

Decomposing the total ΔE_orb_ into pairwise orbital interactions, ΔE_orb(*n*)_, provides valuable insights, particularly regarding the partially occupied orbitals of (HATH_3_)_2_ involved in the bonding. The *trans* and *cis* π-dimers have similar calculated electronic structures, and to simplify the analysis, we focused on the more stable *trans* π-dimer. The deformation densities, which are the difference between the densities of the fragments before and after bond formation, corresponding to ΔE_orb(*n*)_ and the interacting fragment orbitals from the top (middle column) and bottom (rightmost column) HATH_3_ monomers, are depicted in Figure 4. The double arrows (↔) denote the electron sharing between these two monomers. The analysis identifies that the covalent-like bonding interaction can be partitioned into three electron-sharing π-bonding, consisting of one A_1_-symmetric and two degenerate E-symmetric components (ΔE_orb(2,3)_). ΔE_orb(2)_ and ΔE_orb(3)_ contribute 27.9% each to ΔE_orb_. ΔE_orb(1)_ is almost equally strong, contributing 30.0% of ΔE_orb_. This gives an overall triple bond, HATH_3_≡HATH_3_, as the best qualitative description for the bonding situation in the molecule. The NOCV results clearly support the existence of the triple pancake bond between neutral HATH_3_ monomers. Note the multicenter nature of the bond.

**Figure 4:**
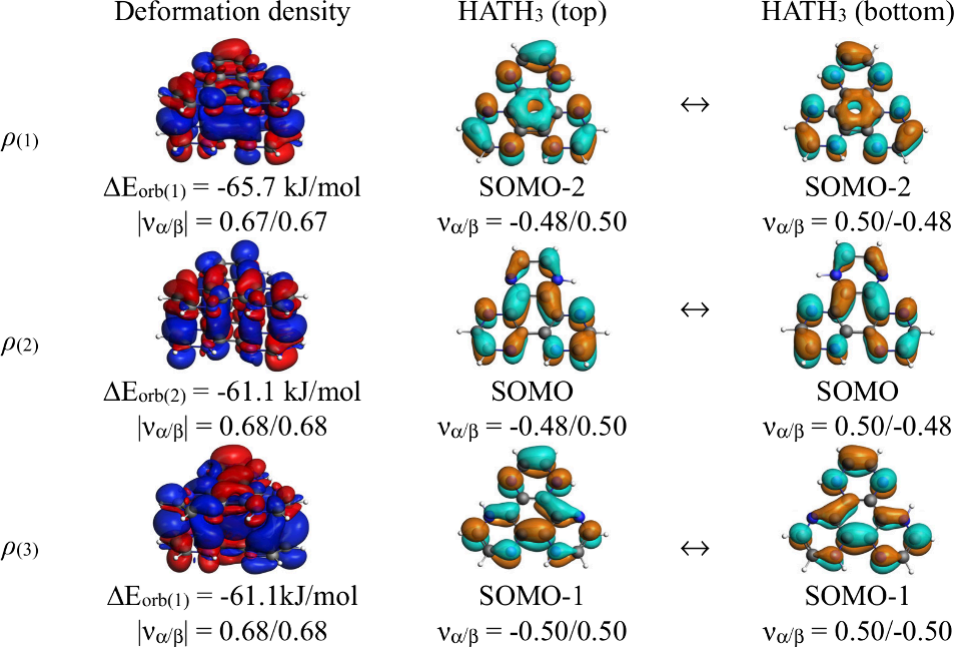
Plot of the deformation densities, Δρ_(n)_ shown as the sum of α and β electronic charge corresponding to ΔE_orb(n)_ and the related interacting orbitals in the (HATH_3_)_2_ π-dimer at the UM06L/TZ2P-ZORA level. The NOVC eigenvalues ν, which are given for the α and β electrons, indicate the quantity of the migrated charge. The direction of the charge flow of the deformation densities is red → blue.

### Examples of Extension of Triply Pancake-Bonded Dimers

By extending the same design strategy to HAT derivatives, the corresponding triradical molecules HATH_3_CN and HANH_3_ were obtained by introducing three hydrogen atoms at nonadjacent nitrogen sites.

The optimized *trans*-cofacial π-dimeric structures of (HATH_3_CN)_2_ and (HANH_3_)_2_ (Figure 5) exhibit relative rotation angles of 7.1° and 8.3°, respectively. Both dimers adopt a concave–concave orientation where each monomer undergoes a subtle out-of-plane deformation. This geometry facilitates exceptionally short vertical separation distances between the central C_6_ rings (2.958 Å for (HATH_3_CN)_2_ and 2.960 Å for (HANH_3_)_2_), which are even more compressed than the same distance in the parent (HATH_3_)_2_ system.

**Figure 5:**
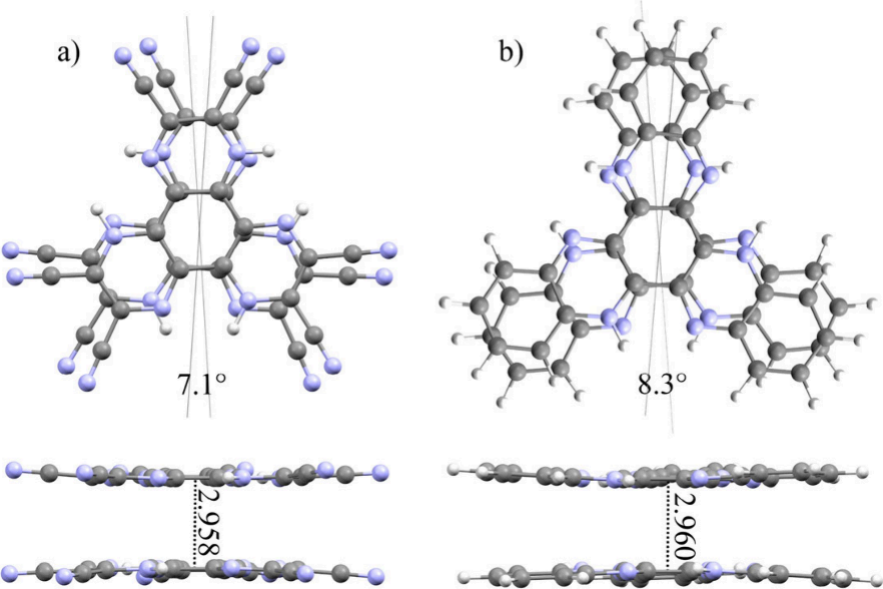
Optimized geometry of two triple pancake bonded π-dimers a) (HATH_3_CN)_2_ and b) (HANH_3_)_2_ adopting a *trans*-cofical arrangement. The vertical separations between the central *C*_6_ rings are expressed in angstroms (Å).

Consistent with these intimate contacts, both π-dimers exhibit large E_int_ of −118.0 and −176.1 kJ/mol, respectively. The large intermolecular bonding interactions arise primarily from the SOMO–SOMO overlap (Table 3). For (HATH_3_CN)_2_, the vdW term remains repulsive at +55.6 kJ/mol, whereas the π-extension in (HANH_3_)_2_ transforms this contribution into an attractive value of −29.7 kJ/mol. This additional dispersion-driven stabilization, coupled with a substantial SOMO–SOMO interaction of −146.4 kJ/mol, accounts for the exceptional bonding strength in the (HANH_3_)_2_ dimer. The calculated bond order, *p*_NO_, remains significantly high at 1.967 for (HATH_3_CN)_2_ and 1.853 for (HANH_3_)_2_. The corresponding FMOs and NOONs for (HATH_3_CN)_2_ and (HANH_3_)_2_ π-dimers are detailed in Figure S7 and S8. We further performed EDA-NOCV analyses on the larger substituted dimer systems, namely (HANH_3_CN)_2_, (HANH_3_)_2_, and (HAN)_2_^6–^ derived from [{ThCl_2_(THF)_2_}_3_(μ-HAN)_2_]. The orbital interaction patterns were found to be highly analogous to those of the HATH_3_ dimer, exhibiting three main bonding components with A_1_, E, and E symmetries, respectively, each contributing comparably to the overall interaction energy. These results, shown in Tables S7 and S8, consistently support their description as triple-pancake-bonded systems. Finally, commenting on the nature of pancake bonding in the {MCl_2_(thf)_2_}_3_(μ-HAN)_2_ coordination complexes, it is insightful that the (HAN)_2_^6–^ and the HANH_3_ dimers both display very comparable gaps, as shown in Table S9 and briefly discussed in the SI. This would impart a similar electronic structure and in fact the ground state is quartet and not a doublet as we found in our computations. In contrast, coordination of the Mg counterions strongly lowers the symmetry of [{Mg­(nacnac)}_3_(HAN)] to *C*_1_ and stabilizes a doublet ground state. In this case we speculate that the Mg coordination plays a significant role in pushing the geometry toward a more overlapping and therefore more interacting configuration, eventually switching the order of the nearly degenerate quartet vs doublet state.

**Table 3:** Interaction Energy Components (kJ/mol) and Spin Expectation Values ⟨S^2^⟩ for (HATH_3_CN)_2_ and (HANH_3_)_2_ π-Dimers at the M06L/6-311++G­(2d,2p) Level^a^

	E_int_	E_SOMO–SOMO_	E_vdW_	⟨S^2^⟩
(HATH_3_CN)_2_				
Singlet	–118.0	–173.6	55.6	0.0000
Septet	55.6	0.0	55.6	12.0510
(HANH_3_)_2_				
Singlet	–176.1	–146.4	–29.7	0.0000
Septet	–29.7	0.0	–29.7	12.0505

a Calculations were performed for different spin states at the singlet ground state geometry based on eqs 2–5.

These results demonstrate the generality of the triple pancake bonding motif across HAT-based systems. Therefore, they would be good candidates for further analysis and perhaps synthesis.

## Conclusions

In summary, we have designed a neutral triradical molecule, HATH_3_, and established the existence of an unusual triple pancake bond in its dimer. This multicentered π–π interaction arises from the overlap of three π-type SOMOs on each monomer. In all three presented cases a high spin (quartet) conjugated molecule forms singlet ground state dimers providing an additional spin-quenching mechanism in addition to the more widely occurring σ-bond formation. Through SOMO–SOMO interactions, HATH_3_ forms *trans*- and *cis*-cofacial π-dimers characterized by exceptionally short intermolecular distances (2.97 Å). EDA-NOCV reveals that the interaction is dominated by orbital contributions, with additional stabilization from electrostatic and dispersion effects. These strong intermolecular interactions can be important in aggregate formation and crystal formation. Extension of this study to HATH_3_ derivatives, HATH_3_CN and HANH_3_, demonstrates that the triple pancake bonding motif remains robust across different peripheral substitutions. This discovery expands the concept of pancake bonding from single and double-bonded systems to metal-free triply bonded systems, offering new opportunities for materials design.

## Computational Details

The geometries and vibrational frequencies of HATH_3_, HATH_3_CN, HANH_3_ and their dimers were employed UDFT using the M06L^48^ method with the 6–311++G­(2d,2p)^52^ basis set. Guess = mix keyword was used to generate an initial guess for UDFT calculations of all spin-unrestricted singlets and for the septet states. All these calculations were carried out with the GAUSSIAN16 program package.^60^ The relative energies of HATH_3_, HATH_3_CN, and HANH_3_ monomers with different spins were calculated using strongly contracted *N*-Electron Valence State Second Order Perturbation Theory (SC-NEVPT2)^61^ calculations based on the UM06L-optimized structures. The CASSCF­(6,6) calculations have been performed using the bonding and antibonding orbitals as the active orbital space for (HATH_3_)_2_ π dimers. The ORCA suite of programs was used for the CASSCF and SC-NEVPT2 computations.^62^

## Approximate Separation of the Interaction Energy: vdW and Pancake Bonding Components

Interaction energy (*E*_*int*_) was calculated by taking the difference between the total energy of the dimer and its monomers.

2 $$E_{\mathrm{int}}=E_{\mathrm{dimer}}-2\cdot E_{\mathrm{monomer}}$$

The separation of the vdW and the attractive SOMO–SOMO interaction is essential for the approximate analysis of the interaction energy, *E*_*int*_.^13,55,56,63^ The idea behind this approximation is that the vdW interaction is not very sensitive to the spin state of the dimer, and that in the high spin state of the dimer the bonding and antibonding SOMO–SOMO interactions approximately cancel. Hence, *E*_*int*_ is approximated as the sum of the specific pancake π–π bonding SOMO–SOMO interaction (*E*_SOMO–SOMO_) and the van der Waals (*E*_*vdW*_) term

3 $$E_{\mathrm{int}}\approx E_{\mathrm{SOMO}-\mathrm{SOMO}}+E_{\mathrm{vdW}}$$

The vdW term includes dispersion, Pauli repulsion, and electrostatic interactions. *E*_*vdW*_ is approximated by the interaction energy of the high-spin (HS) state,^31,55^ *E*_*int*_^*HS*^ taken at the same unrelaxed ground state geometry of the singlet. HS is a septet for π-dimers in this study. Computed for the high-spin state taken at the same distance since, in this case, bonding and antibonding interactions derived from the SOMO orbitals approximately cancel.

4 $$E_{\mathrm{vdW}}\approx E_{\mathrm{int}}^{\mathrm{HS}}\;\left(\mathrm{at the geometry of the singlet}\right)$$

The SOMO–SOMO interaction term for the triple pancake bond is then approximated as follows:

5 $$E_{\mathrm{SOMO}-\mathrm{SOMO}}\approx E_{\mathrm{int}}^{\mathrm{LS}}-E_{\mathrm{int}}^{\mathrm{HS}}$$

LS labels the low-spin state, which is a singlet for the π-dimers in this study.

## Energy Decomposition Analysis-Natural Orbitals for Chemical Valence (EDA-NOCV)

Energy decomposition analysis (EDA) along with the natural orbital for chemical valence (NOCV) method^58,59^ was executed using the ADF 2020 program package^64,65^ at the M06L/TZ2P-ZORA//UM06L/6–311++G­(2d,2p) level.^66–68^

In the EDA method, the interaction energy (Δ*E*_*int*_) between two fragments is divided into four energy terms, viz., the electrostatic interaction energy (Δ*E*_*elstat*_), which represents the quasiclassical electrostatic interaction between the unperturbed charge distributions, the Pauli repulsion (Δ*E*_*pauli*_), which originates from the energy change associated with the transformation from the superposition of the unperturbed electron densities of the isolated fragments to the wave function that properly obeys the Pauli principle through explicit antisymmetrization and renormalization of the product wave function, the orbital interaction energy (Δ*E*_*orb*_), which comes from the mixing of orbitals between the monomer fragments, charge transfer and polarization between the isolated fragments, and the dispersion interaction energy (Δ*E*_*disp*_).

Therefore, the interaction energy (Δ*E*_*int*_) between two fragments can be represented as

6 $$\Delta E_{\mathrm{int}}=\Delta E_{\mathrm{elstat}}+\Delta E_{\mathrm{Pauli}}+\Delta E_{\mathrm{orb}}+\Delta E_{\mathrm{disp}}$$

The EDA-NOCV combination allows the partition of Δ*E*_*orb*_ into pairwise contributions of the orbital interactions, which gives important information about bonding. The starting point is the charge deformation density Δρ­(*r*), a term for the difference between the electron densities of the superimposed fragments before and after bond formation. The deformation density Δρ­(*r*) can be expressed in terms of pairs of complementary eigenfunctions (ψ_k_ ψ_–k_) with the eigenvalues υ_k_ and υ_–k_ that possess the same absolute value but opposite sign.

7 $$\Delta \rho \left(r\right)=\underset{k}{\sum }\Delta \rho_{k}\left(r\right)=\underset{k}{\sum }v_{k}\left[-\psi_{-k}^{2}\left(r\right)+\psi_{k}^{2}\left(r\right)\right]$$

The NOCVs ψ_k_ and the associated eigenvalues *v*_*k*_ are obtained through diagonalization of the difference density matrix Δ*P*_μν_ of the system. eq 7 makes it possible to express the total charge deformation Δρ that goes along with the bond formation in terms of pairwise charge contributions Δ*ρ*_*k*_ that come from particular pairs of NOCV orbitals. The total orbital interaction ΔE_orb_ may likewise be derived from pairwise orbital interaction energies Δ*E*_*orb*_^*k*^ that are associated with Δ*ρ*_*k*_:

8 $$\Delta E_{\mathrm{orb}}=\underset{k}{\sum }\Delta E_{\mathrm{orb}}^{k}=\underset{k}{\sum }v_{k}\left[-F_{-k,-k}^{\mathrm{TS}}+F_{k,k}^{\mathrm{TS}}\right]$$

The terms *F*_–*k*,–*k*_^TS^ and *F*_*k*,*k*_^TS^ are diagonal transition-state (TS) Kohn–Sham matrix elements corresponding to NOCVs with the eigenvalues -*v*_*k*_ and *v*_*k*_, respectively. Here, the term “TS” refers to the charge density, which is intermediate between the density of the final molecule AB and the superimposed fragment densities of A and B. The Δ*E*_*orb*_^*k*^ term of a particular type of bond is assigned by visual inspection of the shape of the deformation density,Δ*ρ*_*k*_. The EDA-NOCV makes it possible to estimate the charge donation Δ*ρ*_*k*_, which is associated with the pairwise orbital interactions, and to visualize its spatial extent. The change in the electron density distribution that comes from bond formation between two fragments or intermolecular interactions during a chemical reaction can be quantitatively expressed through the eigenvalues of the NOCVs. For further information about this method, see references.^69–75^

## Supplementary Material



## Notes

Hückel MO levels and occupancies of HATH_3_, HATH_3_CN, and HANH_3_ monomers; frontier molecular orbitals of *cis* (HATH_3_)_2_, (HATH_3_CN)_2_, and (HANH_3_)_2_ π-dimer; structural parameters and rigid PES scans of (HATH_3_)_2_ π-dimers; EDA-NOCV analysis of *trans* (HATH_3_)_2_ π-dimer, comparison of the ground state of [HAN]^3–^ and HANH_3_. (PDF)
