# Supplementary material for: Triple Pancake Bonding in Neutral π‑Conjugated Dimers: a Computational Study
Source: J Am Chem Soc. 2026 Jul 2;148(27):29146–55. doi: 10.1021/jacs.6c07884 (PMC13383717; doi:10.1021/jacs.6c07884)
Supplement: Supplementary file 1 [file ja6c07884_si_001.pdf]

Supplementary Information

Triple Pancake Bonding in Neutral π-Conjugated Dimers: a Computational Study

Li-juan Cui,[a] Miklos Kertesz,*[b] Zhong-hua Cui*[a,c]

[a]*Institute of Atomic and Molecular Physics, Jilin University, Changchun 130023, China*

E-mail: zcui@jlu.edu.cn

[b]*Department of Chemistry and Institute of Soft Matter, Georgetown University, 37th and O Streets, NW, Washington, DC 20057, USA*

E-mail: kertesz@georgetown.edu

[c]*Key Laboratory of Physics and Technology for Advanced Batteries (Ministry of Education), Jilin University, Changchun 130023, China*

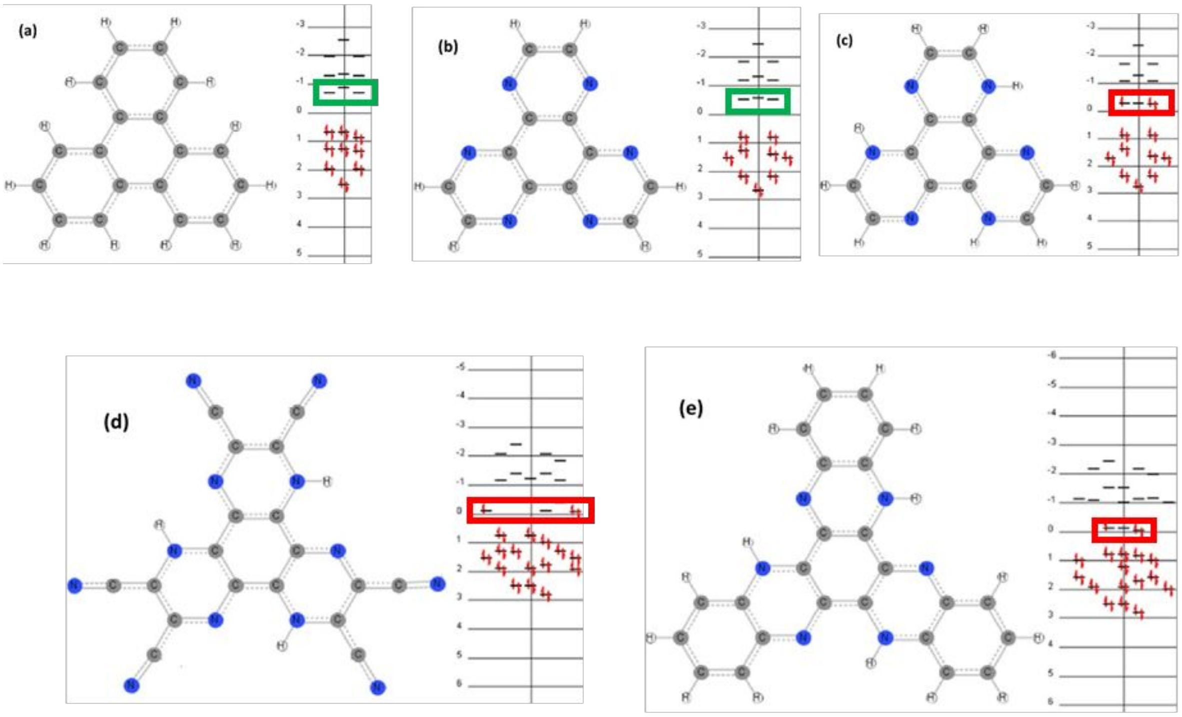

Figure S1. Hückel MO levels and occupancies of (a) TPh, (b) HAT, (c) $HATH_3$, (d) $HATH_3CN$, (e) $HANH_3$. Square symbols indicate the nearly degenerate energy levels. Green: unoccupied, red: occupied levels. The energy scale is the usual β units. Note that the gap between the A and E energy levels is the largest for the parent TPh and significantly smaller for the others.

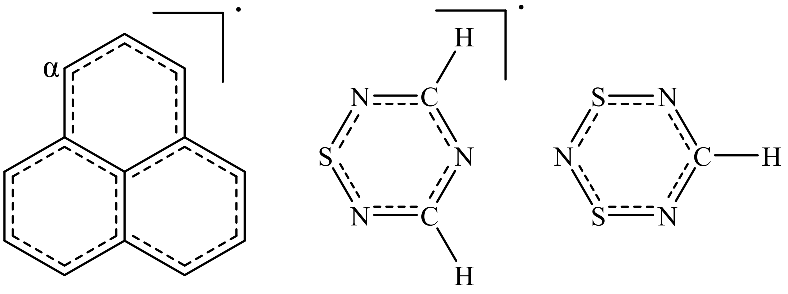

PLY TTA DTTA

Figure S2. Three monomers of pancake-bonded π-systems form the basis of the presented UDFT validation: PLY, TTA, and DTTA.

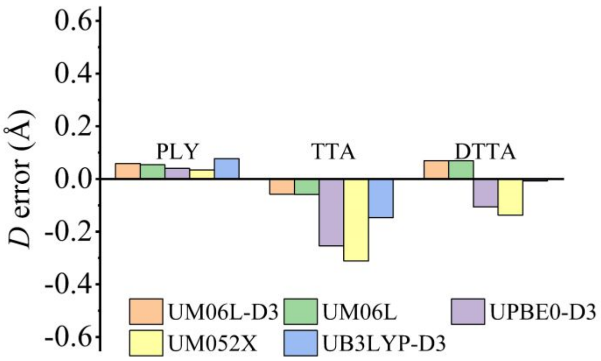

Figure S3. Errors in intermolecular distance for three π-dimers obtained from different UDFT methods with the 6-311++G(2d,2p) basis. The errors are defined as $D_{error} = D_{calcu.} - D_{ref.}$, where $D_{calcu.}$ denotes the UDFT results and $D_{ref.}$ denotes the MR-AQCC reference values. D represents C_α-C_α contacts for 1 and S-S contacts for the other two dimers.

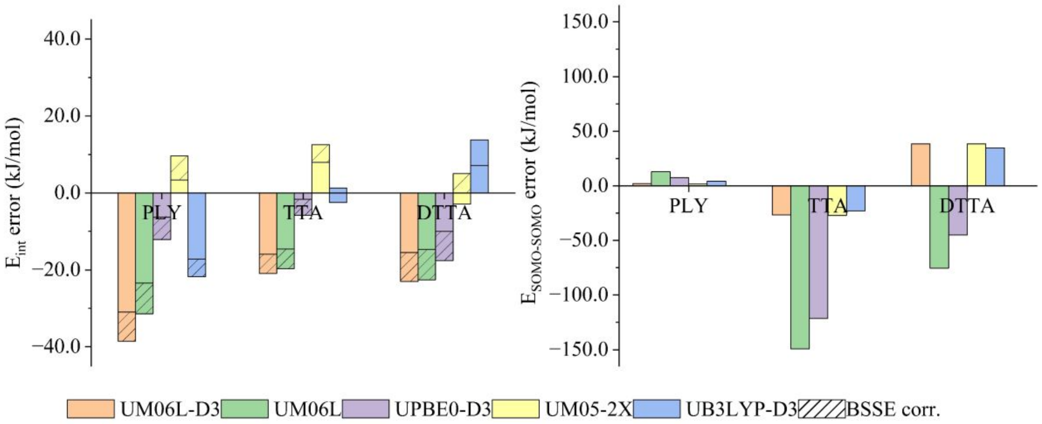

Figure S4. Total interaction energy (E_{int}) and SOMO-SOMO interaction energy ($E_{SOMO\text{-}SOMO}$) errors for three π-dimers obtained from different UDFT methods with the 6-311++G(2d,2p) basis. The errors are defined as $E_{error} = E_{calcu.} - E_{ref.}$, where $E_{calcu.}$ denotes the UDFT results and $E_{ref.}$ denotes the MR-AQCC reference values. Shaded areas in the interaction energy plots indicate BSSE corrections.

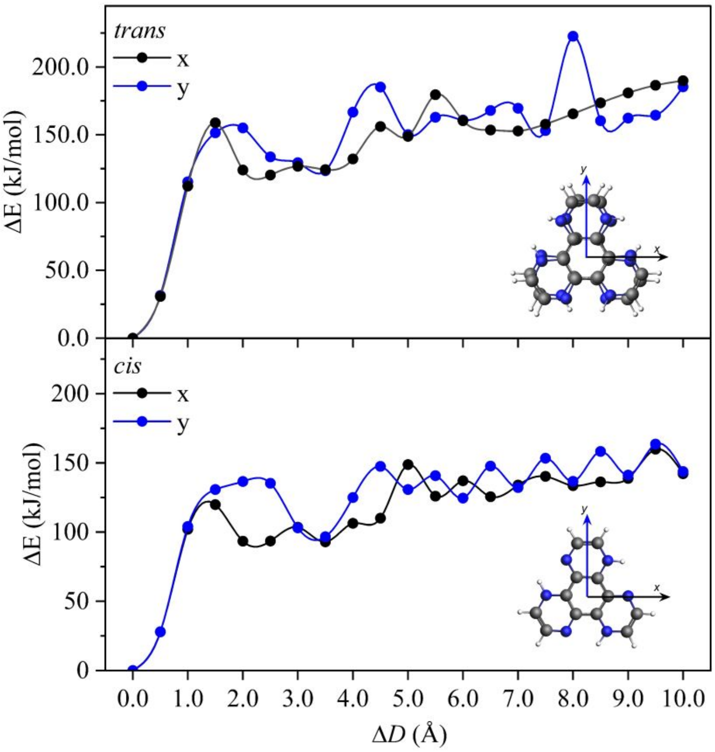

Figure S5. Rigid potential energy surface scan for *trans* and *cis* $(HATH_3)_2$ π-dimer. The curves depict the relative energy (ΔE, kJ/mol) as a function of displacement (Δ*D*, Å) along the x and y axes while maintaining the equilibrium interplanar distance at UM06L/6-311++G(2d,2p) level. The step size of displacement distance in the scan is 0.5 Å.

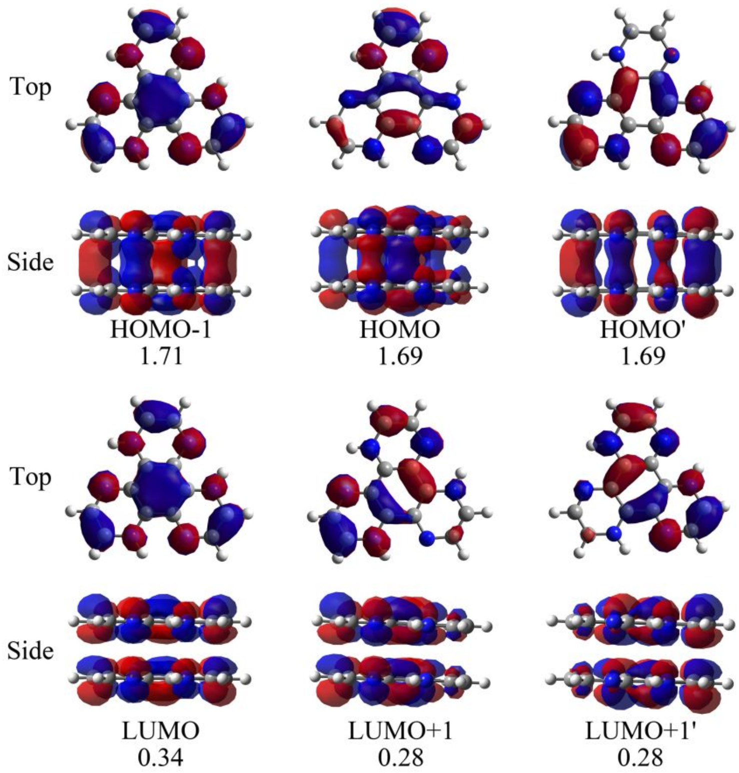

Figure S4. Frontier molecular orbitals of the *cis* $(HATH_3)_2$ π-dimer. Each orbital describing the pancake triple bonds is shown top-down and side-on. NOON values are also shown.

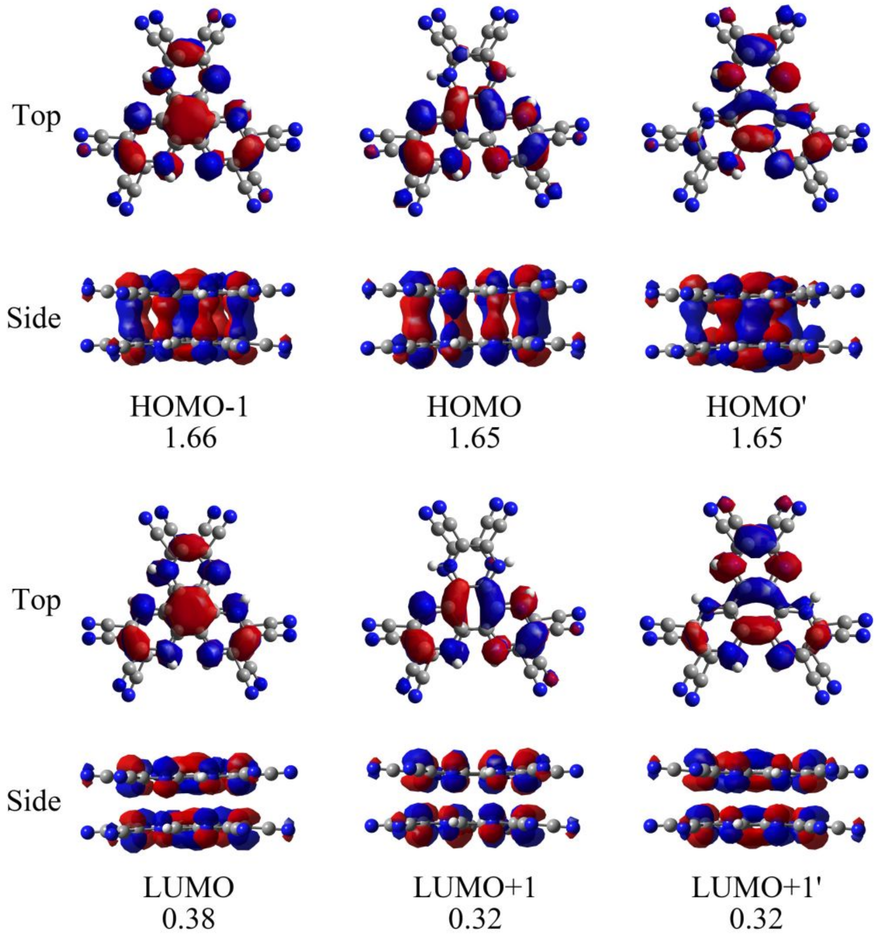

Figure S5. Frontier molecular orbitals of $(HATH_3CN)_2$ π-dimer. Each orbital describing the pancake triple bonds is shown top-down and side-on. NOON values are also shown.

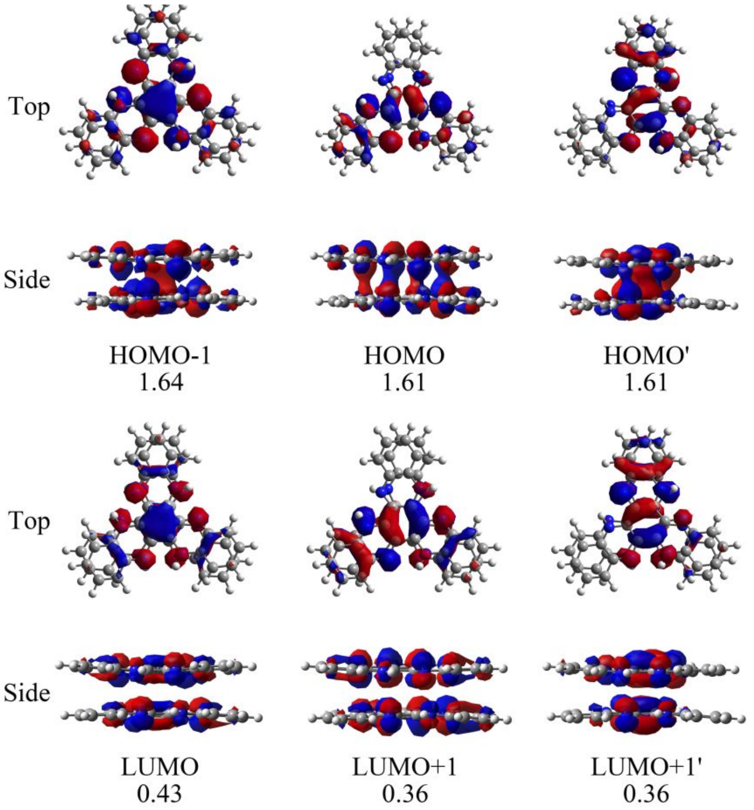

Figure S6. Frontier molecular orbitals of $(HANH_3)_2$ π-dimer. Each orbital describing the pancake triple bonds is shown top-down and side-on. NOON values are also shown.

Table S1. Relative energies (in kJ/mol) and electron configurations in different spin states for the monomers $HATH_3$, $HATH_3CN$, and $HANH_3$ at the NEVPT2/6-311++G(2d,2p) level.

	NEVPT2(3,3)			NEVPT2(3,6)		
State	ΔE	Configuration	Weight	ΔE	Configuration	Weight
			$HATH_3$			
$^4A''$	0.0	111	1.00	0.0	111000	1.00
$^2A''$	16.7	210	0.68	16.7	210000	0.69
		012	0.31		012000	0.29
			$HATH_3CN$			
$^4A''$	0.0	111	1.00	0.0	111000	0.99
$^2A''$	18.0	210	0.72	13.8	210000	0.72
		012	0.27		012000	0.26
			$HANH_3$			
$^4A''$	0.0	111	1.00	0.0	111000	1.00
$^2A''$	43.9	210	0.76	40.6	210000	0.77
		012	0.23		012000	0.21

Table S2. Interaction Energies, E_{int}, and their components, E_{vdW} and $E_{SOMO–SOMO}$ for selected three π-dimers at MR-AQCC level. Data for PLY_2 are from ref. 10, for TTA_2 and $DTTA_2$ from ref. 28. Energy values are given in kJ/mol.

species	*D* (Å)	E_{int}	E_{vdW}	$E_{SOMO–SOMO}$
PLY_2	3.104	-48.1	23.8	-72.0
TTA_2	2.870	-29.3	49.4	-78.7
$DTTA_2$	2.571	-115.9	261.5	-377.4

Table S3. Interaction Energies, E_{int}, and their components, E_{vdW} and $E_{SOMO-SOMO}$ for selected three π-dimers at different UDFT levels with 6-311++G(2d,2p) basis. The values in parentheses include the BSSE corrections. Energy values are given in kJ/mol.

species	UM06L-D3	UM05-2X	UPBE0-D3	UM06L	UB3LYP-D3
			E_{int}		
PLY_2	-86.6<br>(-79.1)	-44.8<br>(-38.5)	-60.2<br>(-54.4)	-79.5<br>(-71.5)	-69.9<br>(-65.3)
TTA_2	-50.2<br>(-45.2)	-21.3<br>(-16.7)	-35.1<br>(-31.0)	-49.0<br>(-43.9)	-31.8<br>(-28.0)
$DTTA_2$	-138.9<br>(-131.4)	-118.8<br>(-110.9)	-133.5<br>(-125.9)	-138.5<br>(-130.5)	-108.8<br>(-102.1)
			$E_{SOMO-SOMO}$		
PLY_2	-69.9	-59.0	-64.4	-70.3	-67.8
TTA_2	-105.4	-228.0	-200.0	-105.9	-101.7
$DTTA_2$	-338.9	-452.7	-422.6	-338.9	-342.7
			E_{vdW}		
PLY_2	-17.2<br>(-9.2)	14.6<br>(20.9)	4.2<br>(10.0)	-9.2<br>(-1.7)	-2.1<br>(2.5)
TTA_2	55.2<br>(60.2)	206.7<br>(211.3)	164.8<br>(169.0)	56.9<br>(61.5)	69.9<br>(73.6)
$DTTA_2$	200.0<br>(207.9)	333.9<br>(341.8)	288.7<br>(296.6)	200.4<br>(208.4)	234.3<br>(241.0)

Table S1. Selected intermolecular distances (Å) of the *trans* and *cis* $(HATH_3)_2$ π-dimers obtained using the UM06L and UM05-2X methods with the 6-311++G(2d,2p) basis set.

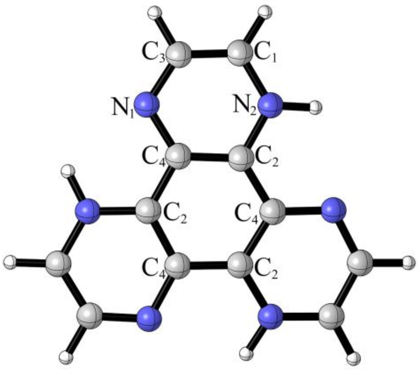

trans			*cis*		
	UM06L	UM05-2X		UM06L	UM05-2X
N_1-N'_2	2.947	2.986	N_1-N'_1	3.004	3.242
C_1-C'_3	2.959	3.019	C_1-C'_1	2.925	3.219
C_2-C'_4	2.968	2.992	N_2-N'_2	2.936	3.187
torsional angle	8.0°	9.4°	C_2-C'_2	2.955	3.186
			C_3-C'_3	2.995	3.287

	C_4-C'_4	2.980	3.201

Table S5. Computed interaction energies, E_{int}, and its components, E_{vdW} and $E_{SOMO-SOMO}$ of the *trans* $(HATH_3)_2$ π-dimer at different UDFT levels with 6-311++G(2d,2p) basis at UM06L/6-311++G(2d,2p) geometry. Energy values are given in kJ/mol.

	UM06L-D3	UM06L	UM05-2X	UPBE0-D3	UB3LYP-D3
E_{int}	-169.5	-158.6	-43.1	-96.7	-120.1
$E_{SOMO-SOMO}$	-212.5	-213.8	-120.9	-156.5	-177.4
E_{vdW}	43.1	55.2	77.8	59.8	57.7

Table S6. The EDA results of the $(HATH_3)_2$ π-dimer calculated at the UPBE0-D3/TZ2P-ZORA level using the UM06L/6-311++G(2d,2p) geometry, considering the interaction between two neutral $HATH_3$ in the quartet state. Energy values are given in kJ/mol.

Energy	Interaction	*trans*	*cis*
ΔE_{int}		-92.9	69.1
ΔE_{hybrid}[a]		0.4	0.4
ΔE_{Pauli}		336.0	314.9
ΔE_{disp}[b]		-88.3 (20.6%)	-86.3 (22.5%)
ΔE_{elstat}[b]		-176.1 (41.1%)	-139.5 (36.3%)
ΔE_{orb}[b]		-164.4 (38.3%)	-158.2 (41.2%)
$\Delta E_{orb(1)}$[c]	electron-sharing π bond (A_1)	-47.0 (28.6%)	-44.8 (28.3%)
$\Delta E_{orb(2)}$[c]	electron-sharing π bond (E)	-45.3 (27.5%)	-44.8 (28.3%)
$\Delta E_{orb(3)}$[c]	electron-sharing π bond (E)	-45.3 (27.5%)	-44.8 (28.3%)
$\Delta E_{orb(rest)}$[c]		-26.9 (16.3%)	-23.8 (15.1%)

[a]Metahybrid correction towards orbital interaction.
[b]The percentage contribution with respect to total attraction is given in parentheses;
[c]The percentage contribution in parentheses is given with respect to total orbital interaction.

Table S7. The EDA results of the $[(HAN)_2]^{6-}$ π-dimer calculated at the UM06L/TZ2P-ZORA level, considering the interaction between two neutral monomer molecules in the quartet state. The geometries of the $[HAN]^{6-}$ π-dimer extracted from the crystal structure of the $[\{ThCl_2(THF)_2\}_3(\mu\text{-}HAN)_2]$. Energy values are given in kJ/mol.

Energy	Interaction	$[(HAN)_2]^{6-}$
ΔE_{int}		1839.3
ΔE_{hybrid}[a]		94.5
ΔE_{Pauli}		269.9
ΔE_{elstat}[b]		1959.1
ΔE_{orb}[b]		-389.7
$\Delta E_{orb(1)}$[c]	electron-sharing π bond (A_1)	-83.5 (21.4%)
$\Delta E_{orb(2)}$[c]	electron-sharing π bond (E)	-83.0 (21.3%)
$\Delta E_{orb(3)}$[c]	electron-sharing π bond (E)	-83.0 (21.3%)
$\Delta E_{orb(rest)}$[c]		-140.2 (36.0%)

[a]Metahybrid correction towards orbital interaction.
[b]The percentage contribution with respect to total attraction is given in parentheses;
[c]The percentage contribution in parentheses is given with respect to total orbital interaction.

Table S8. The EDA results of the $(HATH_3CN)_2$ and $(HANH_3)_2$ π-dimer calculated at the UM06L/TZ2P-ZORA level using the UM06L/6-311++G(2d,2p) geometry, considering the interaction between two neutral monomer molecules in their ground state, which is a quartet. Energy values are given in kJ/mol.

Energy	Interaction	$(HATH_3CN)_2$	$(HANH_3)_2$
ΔE_{int}		-107.9	-163.2
ΔE_{hybrid}[a]		100.8	95.4
ΔE_{Pauli}		143.1	155.6
ΔE_{elstat}[b]		-36.4 (14.5%)	-135.6 (32.7%)
ΔE_{orb}[b]		-214.6 (85.5%)	-183.7 (67.3%)
$\Delta E_{orb(1)}$[c]	electron-sharing π bond (A_1)	-55.2 (25.7%)	-48.5 (26.4%)
$\Delta E_{orb(2)}$[c]	electron-sharing π bond (E)	-51.5 (24.0%)	-43.8 (23.8%)
$\Delta E_{orb(3)}$[c]	electron-sharing π bond (E)	-51.5 (24.0%)	-43.8 (23.8%)
$\Delta E_{orb(rest)}$[c]		-56.4 (26.3%)	-47.6 (23.8%)

[a]Metahybrid correction towards orbital interaction.
[b]The percentage contribution with respect to total attraction is given in parentheses;
[c]The percentage contribution in parentheses is given with respect to total orbital interaction.

Analogy between the isolectronic trianionic $[HAN]^{3-}$ and $HANH_3$.

We computed the trianionic $[HAN]^{3-}$ species in the absence of Mg counterions, based on the [{Mg(nacnac)}$_3$(HAN)] species,[1] at the NEVPT2(3,3)/6-311G(d,p) level of theory. The isolated $[HAN]^{3-}$ fragment was found to exhibit a quartet ground state with D_{3h} symmetry, which is marginally more stable than the corresponding doublet state with C_{2v} symmetry by 1.4 kJ/mol.

It is interesting to note that the partly filled SOMO energy levels in all four cases are nearly degenerate as shown in Table S9 confirming the expectation that the quartet should be the ground state for a four monomer.

Table S9. The A_1 and E singly occupied molecular orbital (SOMO) energy levels (in hartrees) at the Hartree Fock level.

	A_1	E	gap
$HATH_3$	-0.13958	-0.12382	-0.01576
$HATH_3CN$	-0.22608	-0.21294	-0.01314
$HANH_3$	-0.15709	-0.13615	-0.02094
$[HAN]^{3-}$	0.14767	0.17048	-0.02281

In contrast, coordination of the Mg counterions strongly lowers the symmetry of [{Mg(nacnac)}$_3$(HAN)] to C_1 and stabilizes a doublet ground state.[1]

The bridging metal centers in the [{MCl_2(thf)$_2$}$_3$(μ-HAN)$_2$] complexes significantly reduce the intermolecular separation to ~2.7 Å, compared with ~3.0 Å in the $HANH_3$ dimer. In addition, the metal bridges enforce an almost perfectly atom-to-atom cofacial arrangement of the monomeric units, in contrast to the slight rotational offset observed in the $HANH_3$ dimer. Consequently, the combination of the shortened intermolecular distance and the nearly ideal cofacial stacking substantially enhances the SOMO-SOMO orbital overlap, leading to significantly stronger SOMO-SOMO interaction energies in [{MCl_2(thf)$_2$}$_3$(μ-HAN)$_2$].

While this analysis is incomplete, it indicates the subtle interplay of geometry and coordination affecting the preference for one spin state compared to another.

References

[1] Moilanen, J.O., Day, B.M., Pugh, T. and Layfield, R. A., 2015. Open-shell doublet character in a hexaazatrinaphthylene trianion complex. *Chemical Communications*, **2015**, *51*, 11478.
